# Identification of Core Prognosis-Related Candidate Genes in Cervical Cancer via Integrated Bioinformatical Analysis

**DOI:** 10.1155/2020/8959210

**Published:** 2020-03-11

**Authors:** Jianxia Wei, Yang Wang, Kejian Shi, Ying Wang

**Affiliations:** ^1^Department of Obstetrics, Beijing Obstetrics and Gynecology Hospital, Capital Medical University, Beijing 100026, China; ^2^State Key Laboratory of Stem Cell and Reproductive Biology, Institute of Zoology, Chinese Academy of Sciences, Beijing 100101, China; ^3^Beijing Research Institute of Traumatology and Orthopaedics, Beijing 100035, China

## Abstract

**Purposes:**

Cervical cancer (CC) is one of the highest frequently occurred malignant gynecological tumors with high rates of morbidity and mortality. Here, we aimed to identify significant genes associated with poor outcome. *Materials and methods*. Differentially expressed genes (DEGs) between CC tissues and normal cervical tissues were picked out by GEO2R tool and Venn diagram software. Database for Annotation, Visualization and Integrated Discovery (DAVID) was performed to analyze gene ontology (GO) and Kyoto Encyclopedia of Gene and Genome (KEGG) pathway. The protein-protein interactions (PPIs) of these DEGs were visualized by Cytoscape with Search Tool for the Retrieval of Interacting Genes (STRING). Afterwards, Kaplan-Meier analysis was applied to analyze the overall survival among these genes. The Gene Expression Profiling Interactive Analysis (GEPIA) was applied for further validation of the expression level of these genes.

**Results:**

The mRNA expression profile datasets of GSE63514, GSE27678, and GSE6791 were downloaded from the Gene Expression Omnibus database (GEO). In total, 76 CC tissues and 35 normal tissues were collected in the three profile datasets. There were totally 73 consistently expressed genes in the three datasets, including 65 up-regulated genes and 8 down-regulated genes. Of PPI network analyzed by Molecular Complex Detection (MCODE) plug-in, all 65 up-regulated genes and 4 down-regulated genes were selected. The results of the Kaplan-Meier survival analysis showed that 3 of the 65 up-regulated genes had a significantly worse prognosis, while 3 of the 4 down-regulated genes had a significantly better outcome. For validation in GEPIA, 4 of 6 genes (PLOD2, ANLN, AURKA, and AR) were confirmed to be significantly deregulated in CC tissues compared to normal tissues.

**Conclusion:**

We have identified three up-regulated (PLOD2, ANLN, and AURKA) and a down-regulated DEGs (AR) with poor prognosis in CC on the basis of integrated bioinformatical methods, which could be regarded as potential therapeutic targets for CC patients.

## 1. Introduction

Cervical cancer (CC) is the fourth most common female cancer affecting a majority of women worldwide; it is also the leading cause of cancer-associated death in women and around 87% CC-related death occur in the developing world, including China [1]. The high incidence of CC is due to human papilloma virus infection, tobacco smoking, genetic alterations, and other factors. The known genetic alterations related with CC involve the epidermal growth factor receptor (EGFR) [2], human telomerase RNA component (hTERC) [3], phosphatase and tensin homolog (PTEN) [4], c-MYC [5], and other aberrations. However, the mechanism underlying the development of CC is still far from clear. The current therapy for CC mainly includes surgical treatment, cytotoxic chemotherapy, and radiotherapy. Despite advances in these traditional and newly emerging therapeutic modalities for CC, the 5-year disease-free survival (DFS) rates for advanced staged CC patients are only 45% [6]. Hence, it is crucial to investigate and identify the molecular aberrations in CC so as to develop more effective therapeutic strategies.

Gene expression profiling combined with bioinformatical analysis has been frequently used to better understand the molecular mechanisms of diseases, especially cancers. For instance, using a comprehensive bioinformatics analysis, Xia et al. [7] demonstrated that ANLN was dramatically up-regulated in CC tissues, where it predicts poor prognosis. Dai et al. [8] identified that the lower expression of KLF4 and ESR1 is closely related to the poor prognosis of patients with CC. Therefore, analyzing the gene expression profiles and the interaction of differentially expressed genes (DEGs) network of CC tissues is vital for understanding the molecular mechanisms of the causes and pathogenesis of CC and the identification of new prognostic biomarkers that may be exploited therapeutically. However, more work needs to be done to uncover the underlying molecular mechanisms in CC.

In the present study, we analyzed gene expression profiles of GSE63514, GSE27678, and GSE6791 with the GEO2R supported by Gene Expression Omnibus (GEO) and Venn diagram software to obtain the common DEGs, which were subsequently subjected to Database for Annotation, Visualization and Integrated Discovery (DAVID) and classified through the gene ontology (GO) and Kyoto Encyclopedia of Gene and Genome (KEGG) pathway enrichment analysis. Then, we established protein-protein interaction (PPI) network to investigate the protein interaction among the DEGs and performed Cytoscape MCODE to identify core genes among the DEGs. Furthermore, the core DEGs were subjected to the Kaplan Meier plotter online database to assess the significant effect of the genes on CC prognosis (*P* < 0.05) and further validated for the expression level between CC tissues and normal cervical tissues through Gene Expression Profiling Interactive Analysis (GEPIA) (*P* < 0.05). Finally, four DEGs (PLOD2, ANLN, AURKA, and AR) were identified and confirmed to be significantly deregulated in CC tissues compared to normal tissues. Our study may provide some additional useful biomarkers which could be promising and effective targets for diagnosis, prognosis, and drug design of CC.

## 2. Materials and Methods

### 2.1. Microarray Data

We obtained the gene expression profiles of GSE63514, GSE27678, and GSE6791 in CC specimen and normal cervical specimen from NCBI-GEO (https://www.ncbi.nlm.nih.gov/geo), which is a public repository containing microarray-based gene expression profiles. Microarray datasets of GSE63514, GSE27678, and GSE6791 were all based upon the GPL570 Platforms ([HG-U133_Plus_2] Affymetrix Human Genome U133 Plus 2.0 Array) which included 28 CC tissues and 24 normal cervical tissues, 28 CC tissues and 3 normal cervical tissues, and 20 CC tissues and 8 normal cervical tissues, respectively.

### 2.2. Gene Expression Profile Analysis

DEGs between CC tissues and normal cervical tissues were identified by the use of GEO2R online tools with ∣logFC∣ > 1.5 and adjust *P* value < 0.05. The Venn software online (http://bioinformatics.psb.ugent.be/webtools/Venn/) was used to detect the commonly DEGs among the three datasets. The DEGs with logFC > 1.5 were considered as significantly up-regulated genes, while the DEGs with logFC<−1.5 were considered as significantly down-regulated genes.

### 2.3. Gene Ontology and Pathway Analysis

DAVID (https://david.ncifcrf.gov/) is a website bioinformatic database that is designed to identify the biological functions of a considerable number of genes or proteins. GO is a commonly recognized and standardized classification system for defining unique biological functions of genes and its RNA or protein product obtained from high-throughput genome or transcriptome analysis. KEGG is a collection of five manually curated databases dealing with genomes, biological pathways, diseases, drugs, and chemical substrates. DAVID was performed to analyze the enrichment of GO and KEGG pathways of DEGs (*P* < 0.05).

### 2.4. Protein-Protein Interaction (PPI) Analysis

Search Tool for the Retrieval of Interacting Genes (STRING) is an online database for evaluation of PPIs. To investigate the potential protein correlations among these DEGs, STRING was applied and interactions with combined score ≥0.4 (medium confidence) were considered significant. Furthermore, Cytoscape was performed to visualize the interaction network. The Molecular Complex Detection (MCODE) plug-in was used to check modules of the PPI network.

### 2.5. Survival Analysis and RNA Sequencing Expression of Hub Genes

Kaplan-Meier plotter is a web-accessible tool commonly used for assessing the effect of a huge number of genes on survival on the basis of EGA, TCGA database, and GEO (Affymetrix microarrays only). The log rank *P* value and hazard ratio (HR) with 95% confidence intervals were computed and showed on the plot. To validate the expression of these DEGs, the Gene Expression Profiling Interactive Analysis (GEPIA) website was applied to analyze the data of RNA sequencing expression based on thousands of samples from the GTEx projects and TCGA.

## 3. Results

### 3.1. Identification of DEGs in Cervical Cancers

**Figure 1:**
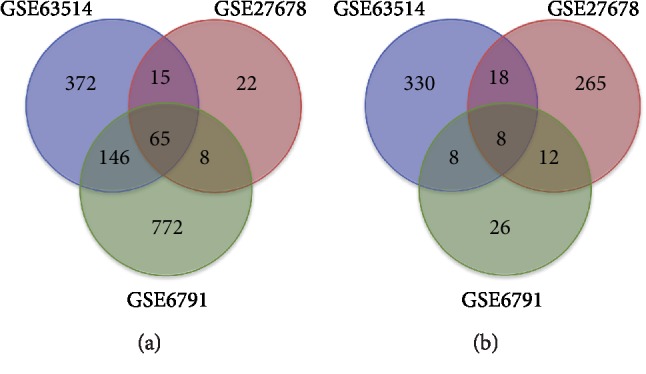
Authentication of 73 common DEGs in the three datasets (GSE63514, GSE27678, and GSE6791) via Venn diagrams software. Different color represents different datasets. (a) 65 DEGs were commonly up-regulated in the three datasets (logFC > 1.5). (b) 8 DEGs were commonly down-regulated in the three datasets (logFC<−1.5).

To identify genes that are closely related to CC prognosis, first of all, we sought to explore DEGs that are possibly involved in the progression from normal cervical epithelium tissue to CC. We collected raw data from different series (GSE63514, GSE27678, and GSE6791) to increase the sample size. Three datasets totally included 76 CC tissues and 35 normal cervical tissues. These raw microarray datasets were normalized data, which is shown in Supplementary [Supplementary-material supplementary-material-1]. By use of the GEO2R online tools, we extracted 1175, 524, and 1179 DEGs from microarray datasets of GSE63514, GSE27678, and GSE6791, respectively. Subsequently, the Venn diagram software was utilized to obtain DEGs commonly present in all the three datasets. As can be seen from Figure 1 and Table 1, a total of 73 commonly deregulated DEGs were identified, including 65 up-regulated genes (logFC > 1.5) and 8 down-regulated genes (logFC<−1.5) in the CC tissues compared with normal tissues.

### 3.2. Gene Ontology and KEGG Pathway Analysis of DEGs in Cervical Cancers

Secondly, we explored the GO and KEGG pathway of the identified DEGs. To gain this goal, all 73 commonly deregulated DEGs were analyzed by DAVID web tool and the results of the GO analysis indicated that (1) for biological processes (BP), the up-regulated DEGs were particularly enriched in regulation of DNA replication, cell division, mitotic nuclear division, G1/S transition of mitotic cell cycle, sister chromatid cohesion, and DNA replication initiation, and the down-regulated DEGs in positive regulation of cell proliferation, positive regulation of cell differentiation, and negative regulation of epithelial cell proliferation; (2) for cell component (CC), the up-regulated DEGs were mostly significantly enriched in the nucleoplasm, nucleus, midbody, condensed chromosome kinetochore, kinetochore, and spindle microtubule. However, the down-regulated DEGs was not significantly enriched in any CC term of category; (3) for molecular function (MF), the up-regulated DEGs were mostly significantly enriched in ATP binding, protein binding, chromatin binding, DNA binding, and single-stranded DNA-dependent ATPase activity, and the down-regulated DEGs in serine-type peptidase activity (Table 2, *P* < 0.05).

As shown in Table 3, the KEGG analysis results demonstrated that the up-regulated DEGs were mostly significantly enriched in cell cycle, DNA replication, fanconi anemia pathway, and p53 signaling pathway, while the down-regulated DEGs was not significantly enriched in any signaling pathways (*P* < 0.05).

### 3.3. Protein-Protein Interaction Network (PPI) and Modular Analysis

**Figure 2:**
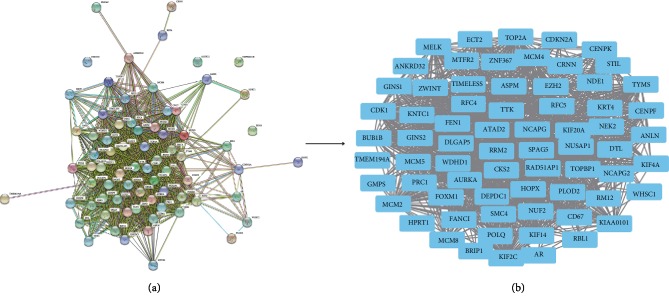
PPI network of common DEGs were constructed by STRING database and Module analysis. (a) There were a total of 73 DEGs in the DEGs PPI network complex. The nodes indicate proteins; the lines represent the interaction of proteins. (b) Module analysis through Cytoscape software.

To explore the PPI networks of the identified DEGs, with a special focus upon hub genes that possibly play key roles in the progression of cervical cancer, a total of 73 DEGs were imported into the DEGs PPI network, which consisted of 69 nodes and 4 edges. A significant module was obtained from the DEGs PPI network using Cytotype MCODE, showing that 69 central nodes (including 4 down-regulated and 65 up-regulated genes) were identified (Figure 2).

### 3.4. Analysis of Key Genes by the Kaplan-Meier Plotter and GEPIA

**Figure 3:**
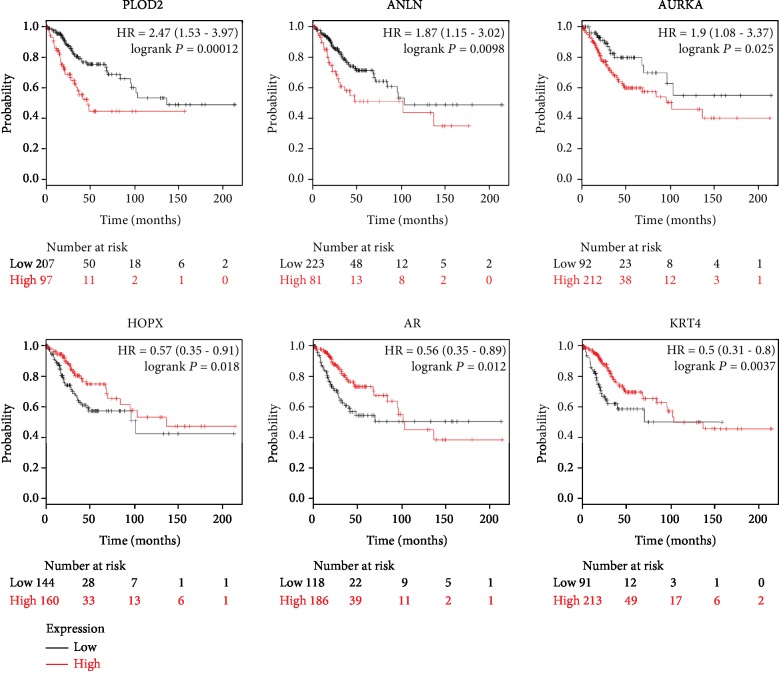
The significant effect of the 6 hub genes on CC prognosis. Kaplan-Meier plotter online tools were performed to identify the prognostic information of the 69 central DEGs and 6 of 69 DEGs had a significantly worse survival rate (*P* < 0.05).

**Figure 4:**
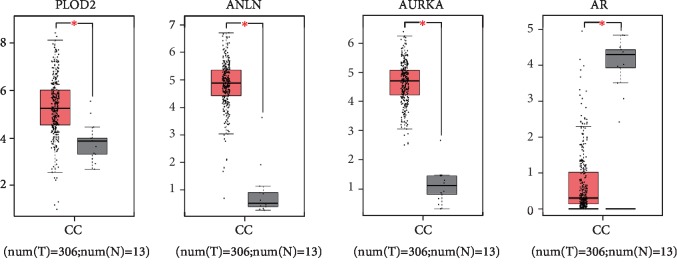
Validation of the expression of 4 hub genes in CC tissues via GEPIA website. Red color represents tumor samples, while gray color represents normal samples.

After the identification of hub genes that might be involved in CC progression, we performed Kaplan-Meier plotter (http://kmplot.com/analysis) to identify 69 hub genes survival data. As observed in Figure 3 3 of the 65 up-regulated genes had a significantly worse survival while 3 of the 4 down-regulated genes had a significantly better outcome (*P* < 0.05). To further validate that the hub genes that are significantly associated with CC prognosis is indeed differentially expressed between normal cervical tissues and CC tissues, GEPIA, a web server for cancer and normal gene expression profiling and interactive analyses, was applied for further confirmation of the expression level of the 6 hub genes (PLOD2, ANLN, AURKA, HOPX, AR, and KRT4) between cancerous and normal tissues. Results showed that all of the three up-regulated genes (PLOD2, ANLN, and AURKA) were proved to be significantly highly expressed, and one of the three down-regulated genes (AR) was confirmed in CC samples compared to normal cervical samples (Figure 4). The expression level of these 4 candidate genes was also verified in a separate GEO dataset GSE64217 (Supplementary [Supplementary-material supplementary-material-1]). Taken together, these data demonstrated that these 4 candidate genes were clearly related to the prognosis of CC patients. Briefly, patients whose tissues displayed a higher expression of each of PLOD2, ANLN, and AURKA or a lower expression of AR had significantly shorter overall survival. The percentage of CC patients that have a worse overall survival is indicated in Supplementary [Supplementary-material supplementary-material-1].

### 3.5. PPI Network of 4 Hub Candidate Genes

**Figure 5:**
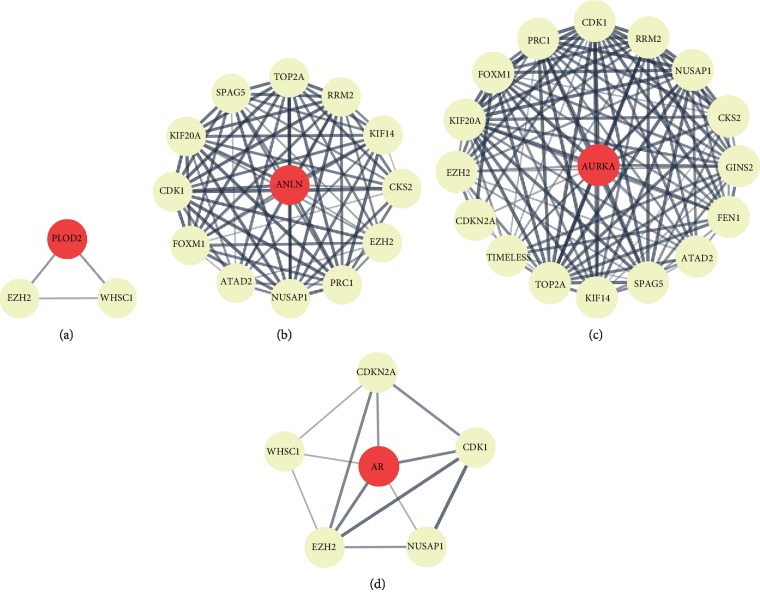
STRING interaction map of each of the 4 hub genes and genes involved in the progression of CC. Many genes involved in the progression of cervical cancer have interactions with (a) PLOD2, (b) ANLN, (c) AURKA, and (d) AR for example EZH2. Line thickness indicates the strength of data supporting interaction.

To explore the possible molecular mechanisms of the 4 hub candidate genes (PLOD2, ANLN, AURKA, and AR) involved in CC progression, the protein-protein interaction (PPI) between each of the 4 hub candidate genes (PLOD2, ANLN, AURKA, and AR) and CC-related DEGs was analyzed. According to the PPI network, many genes involved in CC progression have interactions with these 4 hub genes, for example EZH2, which serves as an interacting protein in common (Figure 5).

## 4. Conclusion

Cervical cancer development is a complex process associated with multiple genetic and environmental factors. Despite numerous researches progress has been made in uncovering the potential molecular mechanism of the development of CC, the underlying mechanism remains unresolved.

To discover more promising and useful prognosis-related biomarkers in CC, the present research performed integrated bioinformatical analysis based upon three mRNA expression profile datasets (GSE63514, GSE27678, and GSE6791). Seventy-six CC specimens and thirty-five normal specimens were enrolled in this study. Using GEO2R and Venn software online, we revealed a total of 73 common DEGs (∣logFC∣ > 1.5 and adjust *P* value < 0.05), including 65 up-regulated genes and 8 down-regulated genes in the CC tissues compared with normal tissues. Subsequently, GO analysis and KEGG pathway enrichment analysis by DAVID method demonstrated that (1) for biological processes (BP), the up-regulated DEGs were particularly enriched in regulation of DNA replication, cell division, mitotic nuclear division, G1/S transition of mitotic cell cycle, sister chromatid cohesion, and DNA replication initiation, and the down-regulated DEGs in positive regulation of cell proliferation, positive regulation of cell differentiation, and negative regulation of epithelial cell proliferation; (2) for cell component (CC), the up-regulated DEGs were mostly significantly enriched in the nucleoplasm, nucleus, midbody, condensed chromosome kinetochore, kinetochore, and spindle microtubule. However, the down-regulated DEGs was not significantly enriched in any CC term of category; (3) for molecular function (MF), the up-regulated DEGs were mostly significantly enriched in ATP binding, protein binding, chromatin binding, DNA binding, and single-stranded DNA-dependent ATPase activity, and the down-regulated DEGs in serine-type peptidase activity (*P* < 0.05). For pathway enrichment analysis, the up-regulated DEGs were mostly significantly enriched in cell cycle, DNA replication, fanconi anemia pathway, and p53 signaling pathway, while the down-regulated DEGs was not significantly enriched in any signaling pathways (*P* < 0.05). Moreover, via the STRING online database and Cytoscape software, DEGs PPIs network analysis revealed 69 central nodes, including 4 down-regulated and 65 up-regulated genes. Next, all 69 central DEGs underwent survival analysis via Kaplan-Meier plotter tool and 3 of the 65 up-regulated genes were shown to be significantly associated with worse overall survival in CC patients, while 3 of the 4 down-regulated genes had a significantly better outcome. Furthermore, by GEPIA analysis, all of the 3 up-regulated genes were proved to be significantly overexpressed, and 1 of the 3 down-regulated genes was confirmed in CC tissues. Finally, based upon PPI network, we identified EZH2 as a common interacting protein for these candidate genes. Thus, these key DEGs and their correlated functions may be implicated in the development of cervical cancer.

Procollagen-lysine, 2-oxoglutarate 5-dioxygenase 2 (PLOD2), also known as BRKS2, LH2, and TLH, is a membrane-bound homodimeric enzyme that specifically catalyzes the hydroxylation of lysyl residues in the telopeptide of procollagens [9]. This enzyme has been involved in the formation of the extracellular matrix and various pathological processes, including epithelial-mesenchymal transition and metastasis. Definitely, the abnormal expression of PLOD2 may contribute to the development and progression of several types of cancer. Overexpression of PLOD2 has been observed in laryngeal squamous cell carcinoma, biliary tract cancer, hepatocellular carcinoma, breast cancer, bladder cancer, sarcoma, oral carcinoma, and renal cell carcinoma and is closely related to a poor prognosis. Thus, PLOD2 has been regarded as a novel prognostic factor in many types of cancer. However, the role of PLOD2 in cervical cancer remains unknown. Here, we found that PLOD2 overexpression was significantly associated with poor clinical prognosis. This evidence indicated that PLOD2 might play a crucial role in cervical cancer progression.

Anillin actin-binding protein (ANLN), also named as FSGS8, has been identified as an actin-binding protein that is associated with actin cytoskeletal dynamics. Previous studies have suggested that ANLN expression is elevated in most human cancers and is associated with poor clinical outcomes in patients with breast [10], bladder [11], colorectal [12], and lung cancers [13]. Recently, Xia et al. [7] reported that ANLN expression correlates with poor clinical outcomes in cervical cancer. Consistent with this finding, our study showed that ANLN overexpression was significantly associated with poor prognosis in cervical cancer, suggesting a potential role in the progression of cervical cancer.

Aurora Kinase B (AURKB) is a key serine/threonine protein kinase that catalyses critical phosphorylation events in mitosis and cytokinesis. Previous studies have shown that AURKB overexpression is associated with tumor progression in renal cell carcinoma [14], lung cancer [15], anaplastic thyroid carcinoma [16], hepatocellular carcinoma [17], and head and neck squamous cell carcinoma [18]. However, the expression and role of AURKB in development and progression of cervical cancer have not been fully explored. In our study, we found that AURKB up-regulation was associated with poor clinical prognosis in cervical cancer, suggesting that AURKB may play a vital role in cervical cancer, and thus may function as a potential therapeutic target for cervical cancer.

Androgen receptor (AR) acts as an androgen-activated transcription factor that regulates eukaryotic gene expression and affects cellular proliferation and differentiation in a wide variety of human adult tissues, including male and female reproductive tissues [19]. A growing amount of literature has demonstrated that AR overexpression is positively correlated with cancer progression and poor prognosis in prostate cancer [20, 21] and colorectal cancer [22]. However, the role of expression alterations of AR in relation to cervical cancer is not well understood. A previous study has shown that AR expression is frequently decreased or lost in advanced cervical cancer versus normal cervix epithelium [23]. Consistently, we found that AR was underexpressed in cervical cancer and was significantly related to poor clinical prognosis, providing insights concerning a potential role of AR in cervical carcinogenesis.

Based on the PPI network, we identified several CC-associated genes that probably have interactions with the above 4 identified candidate genes, especially enhancer of zeste homolog 2 (EZH2), an interacting protein in common for these candidates. EZH2 is one of the histone methyltransferases and has been shown to have a strong adverse relationship with proliferation, clinical and biologic behaviors, and overall survival in CC [24].

To sum up, the current study identified four key genes with prognostic value involved in CC, including PLOD2, ANLN, AURKA, and AR. These genes could be potentially applied in the molecular diagnosis or treatment of CC. However, further investigations are highly required to fully decipher the molecular mechanisms of these genes in CC.

## Figures and Tables

**Table 1:** All 73 commonly differentially expressed genes (DEGs) were detected from three profile datasets, including 65 up-regulated genes and 8 down-regulated genes in the CC tissues compared to normal cervical tissues.

DEGs	Gene names
Up-regulated	TOPBP1 GINS1 ANLN WHSC1 KNTC1 FOXM1 EZH2 CDK1 SMC4 AURKA KIF14 KIF4A NCAPG2 KIF2C TYMS MCM5 MELK ZWINT RFC4 PLOD2 POLQ NUF2 CDC7 CKS2 ECT2 DEPDC1 NDE1 MTFR2 ASPM SPAG5 ATAD2 BRIP1 STIL PRC1 RRM2 TOP2A FEN1 HPRT1 FANCI ZNF367 WDHD1 MCM2 MCM4 BUB1B TIMELESS DLGAP5 RAD51AP1 SLF1 DTL KIF20A CENPK GMPS CDKN2A KIAA0101 TTK MCM8 NCAPG RFC5 NEMP1 RMI2 NEK2 RBL1 GINS2 CENPF NUSAP1

Down-regulated	TMPRSS1B ALOX12 ENDOU KRT4 CRNN EDN3 AR HOPX

**Table 2:** Gene ontology analysis of differentially expressed genes in cervical cancer.

Expression	Category	Term	Count	%	*P* value	FDR
Up-regulated	GOTERM_BP_DIRECT	GO:0006260~DNA replication	17	26.15384615	2.34*E*-19	3.33*E*-16
	GOTERM_BP_DIRECT	GO:0051301~cell division	19	29.23076923	2.73*E*-16	3.22*E*-13
	GOTERM_BP_DIRECT	GO:0007067~mitotic nuclear division	12	18.46153846	1.53*E*-09	2.18*E*-06
	GOTERM_BP_DIRECT	GO:0000082~G1/S transition of mitotic cell cycle	9	13.84615385	3.62*E*-09	5.16*E*-06
	GOTERM_BP_DIRECT	GO:0007062~sister chromatid cohesion	8	12.30769231	9.91*E*-08	1.41*E*-04
	GOTERM_BP_DIRECT	GO:0006270~DNA replication initiation	6	9.230769231	1.09*E*-07	1.55*E*-04
	GOTERM_CC_DIRECT	GO:0005654~nucleoplasm	42	64.61538462	9.29*E*-19	1.04*E*-15
	GOTERM_CC_DIRECT	GO:0005634~nucleus	47	72.30769231	3.90*E*-12	4.37*E*-09
	GOTERM_CC_DIRECT	GO:0030496~midbody	11	16.92307692	2.43*E*-11	2.73*E*-08
	GOTERM_CC_DIRECT	GO:0000777~condensed chromosome kinetochore	9	13.84615385	6.91*E*-10	7.76*E*-07
	GOTERM_CC_DIRECT	GO:0000776~kinetochore	8	12.30769231	1.33*E*-08	1.50*E*-05
	GOTERM_CC_DIRECT	GO:0005876~spindle microtubule	6	9.230769231	4.45*E*-07	4.99*E*-04
	GOTERM_MF_DIRECT	GO:0005524~ATP binding	23	35.38461538	8.92*E*-09	1.03*E*-05
	GOTERM_MF_DIRECT	GO:0005515~protein binding	55	84.61538462	1.75*E*-08	2.03*E*-05
	GOTERM_MF_DIRECT	GO:0003682~chromatin binding	10	15.38461538	1.37*E*-05	0.015903719
	GOTERM_MF_DIRECT	GO:0003677~DNA binding	19	29.23076923	2.60*E*-05	0.030167688
	GOTERM_MF_DIRECT	GO:0043142~single-stranded DNA-dependent ATPase activity	3	4.615384615	6.05*E*-04	0.699450245

Down-regulated	GOTERM_BP_DIRECT	GO:0008284~positive regulation of cell proliferation	3	37.5	0.01471255	15.73085767
	GOTERM_BP_DIRECT	GO:0045597~positive regulation of cell differentiation	2	25	0.015325148	16.33389401
	GOTERM_BP_DIRECT	GO:0050680~negative regulation of epithelial cell proliferation	2	25	0.023116277	23.66719796
	GOTERM_MF_DIRECT	GO:0008236~serine-type peptidase activity	2	25	0.025837913	21.29783341

**Table 3:** KEGG pathway analysis of differentially expressed genes in cervical cancer.

Pathway ID	Name	Count	%	*P* value	Genes
hsa04110	Cell cycle	9	12.32876712	7.44*E*-09	CDC7, CDK1, CDKN2A, RBL1, TTK, BUB1B, MCM2, MCM4, MCM5
hsa03030	DNA replication	6	8.219178082	1.45*E*-07	RFC5, RFC4, MCM2, MCM4, MCM5, FEN1
hsa03460	Fanconi anemia pathway	3	4.109589041	0.015602313	FANCI, BRIP1, RMI2
hsa04115	p53 signaling pathway	3	4.109589041	0.02427143	CDK1, CDKN2A, RRM2

## Data Availability

The data used to support the findings of this study are available from the corresponding author upon reasonable request.

## Supplementary Materials

Supplementary Materials Supplementary Figure 1: normalization of gene expression. (a) The normalization of GSE63514 data, (b) the normalization of GSE27678 data, and (c) the normalization of GSE6791 data. Supplementary Table 1: genome-wide analysis of gene expression in cervical squamous carcinoma compared to that in normal cervical mucosa (GSE64217). Supplementary Table 2: the percentage of CC patients that have a worse overall survival.
