# Supplementary material for: Identification of Core Prognosis-Related Candidate Genes in Cervical Cancer via Integrated Bioinformatical Analysis
Source: Biomed Res Int. 2020 Mar 11;2020:8959210. doi: 10.1155/2020/8959210 (PMC7097776; doi:10.1155/2020/8959210)
Supplement: Supplementary Materials — Supplementary Figure 1: normalization of gene expression. (a) The normalization of GSE63514 data, (b) the normalization of GSE27678 data, and (c) the normalization of GSE6791 data. Supplementary Table 1: genome-wide analysis of gene expression in cervical squamous carcinoma compared to that in normal cervical mucosa (GSE64217). Supplementary Table 2: the percentage of CC patients that have a worse overall survival. [file 8959210.f1.pdf]

Supplementary Figure 1. Normalization of gene expression. (A) The normalization of GSE63514 data, (B) The normalization of GSE27678 data, and (C) The normalization of GSE6791 data.

Supplementary Figure 1

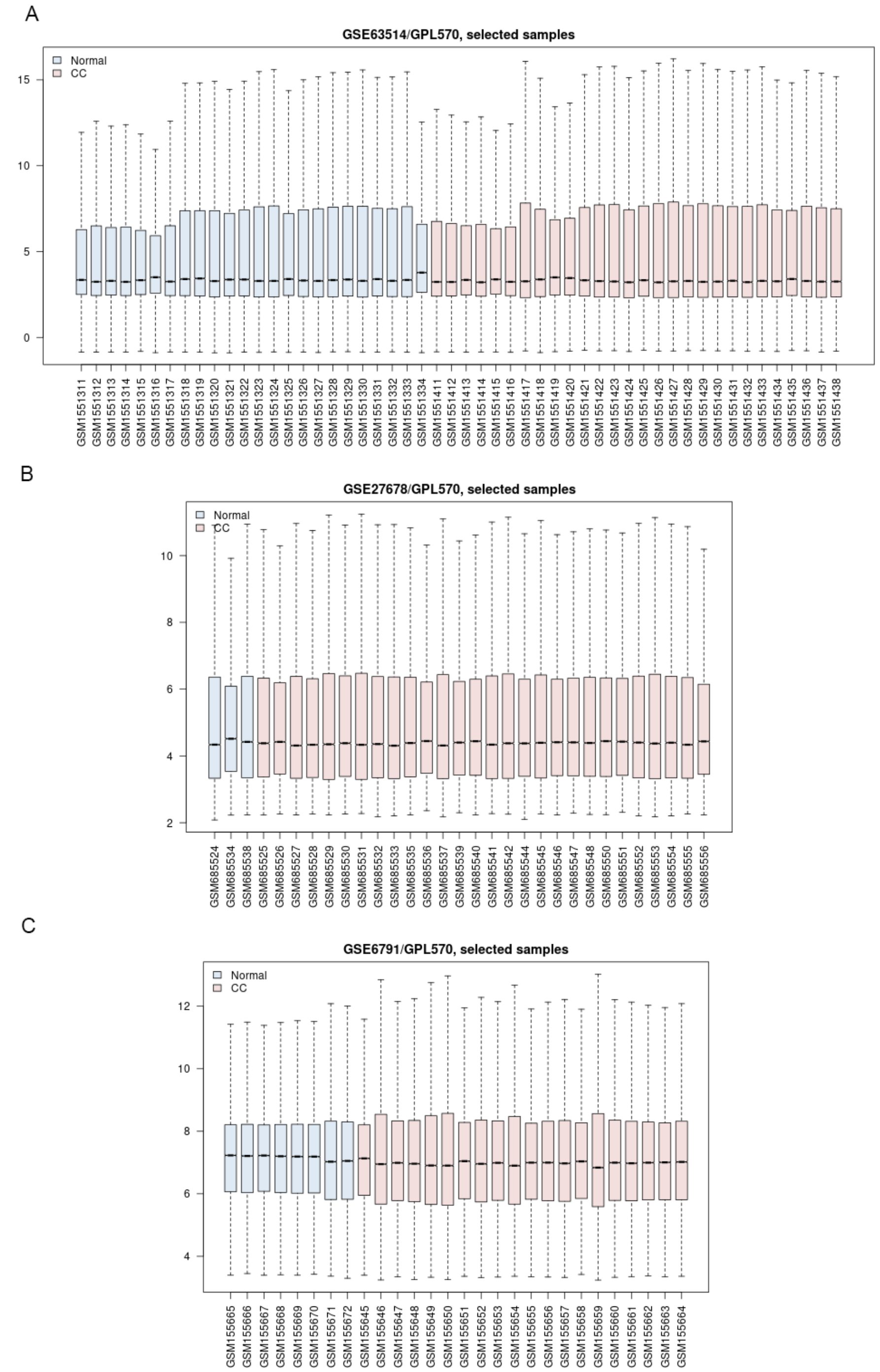

Supplementary Table 1. Genome-wide analysis of gene expression in cervical squamous carcinoma compared to that in normal cervical mucosa (GSE64217)

ID	adj.P.Val	logFC	Gene.symbol	Gene.title
ILMN_1767351	0.0318	-1.47	AR	androgen receptor
ILMN_1771599	0.0143	2.32	PLOD2	procollagen-lysine, 2-oxoglutarate 5-dioxygenase 2
ILMN_1739645	0.016	2.09	ANLN	anillin actin binding protein
ILMN_1680955	0.0146	2.35	AURKA	aurora kinase A

Supplementary Table 2. The percentage of CC patients that have a worse overall survival.

Gene.symbol	Number of patients with high expression level	Number of patients with low expression level	Percentage of patients with worse prognosis
PLOD2	97	207	31.90%
ANLN	81	223	26.60%
AURKA	212	92	69.70%
AR	186	118	38.80%
